# FasParser: a package for manipulating sequence data

**DOI:** 10.24272/j.issn.2095-8137.2017.017

**Published:** 2017-03-18

**Authors:** Yan-Bo Sun

**Affiliations:** ^1^State Key Laboratory of Genetic Resources and Evolution, Kunming Institute of Zoology, Chinese Academy of Sciences, Kunming 650223, China

**Keywords:** FasParser, Batch processing, Sequence comparison, Extraction and filtration

## Abstract

A computer software package called 'FasParser' was developed for manipulating sequence data. It can be used on personal computers to perform series of analyses, including counting and viewing differences between two sequences at both DNA and codon levels, identifying overlapping regions between two alignments, sorting of sequences according to their IDs or lengths, concatenating sequences of multiple loci for a particular set of samples, translating nucleotide sequences to amino acids, and constructing alignments in several different formats, as well as some extracting and filtrating of data for a particular FASTA file. Majority of these functions can be run in a batch mode, which is very useful for analyzing large data sets. This package can be used by a broad audience, and is designed for researchers that do not have programming experience in sequence analyses. The GUI version of FasParser can be downloaded from https://github.com/Sun-Yanbo/FasParser, free of charge.

## INTRODUCTION

Recent developments in sequencing technology function to generate a vast amount of DNA and RNA sequence data. Analyses based on these sequences are one of the most important means of assessing their potential for biological inference. The amount of available sequence data has made their manipulation tricky, especially for researchers without programming experience. Hence, the development of user-friendly software facilitates research using batch modes for sequence extraction, filtration, translation and conversions of file formats.

The program package MEGA (Kumar et al., 1994), which was developed decades ago, has achieved worldwide usage. Although it has manipulation functions, such as sequence viewing and format conversion, it focused mainly on various statistical analyses of molecular evolution. Many sequence manipulations still require manual work or the use of other tools (i.e., Microsoft Office Excel). Examples include the concatenation of loci from multiple sequence files, the extraction of some gene sequences from a whole genome, and the filtering of very short sequences in an alignment. Another package, BioEdit (Hall, 1999), can handle most simple sequence editing and manipulation functions. However, it inefficiently handles batch processing and can only deal with one alignment file at a time.

**Figure 1:**
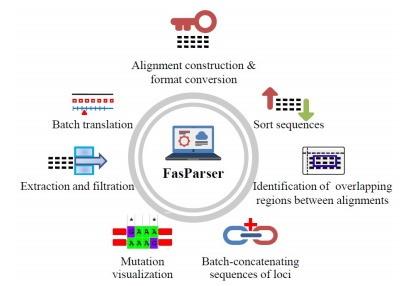
Overview of the functions provided by FasParser

Herein, I provide the new program package 'FasParser' for manipulating sequence files. It has a user-friendly GUI and batch processing modes, which allows users to handle multiple sequence files in a simple way. Presently, the package has seven main programs/functions (Figure 1): (1) counting and viewing the differences between two sequences at the DNA and codon levels; (2) identifying overlapping columns of two alignments of a same gene; (3) sorting sequences according to ID, sequence length, or ID list provided by user; (4) concatenating sequences for a particular set of samples from multiple sequence files; (5) batch-translating protein -coding nucleic acid sequences into amino acids; (6) constructing alignments with different formats; and (7) extracting and filtering sequences according to ID or sequence length. FasParser is a standalone application that has been compiled and tested on Windows 7/10 operating systems. Only available computer memory limits the size of data to be analyzed.

## BATCH PROCESSING

This new package can batch process several commonly used procedures including merging sequences, translating, aligning and converting formats. For merging, it can obtain a "super sequence" by concatenating all the loci sequences for a particular set of samples. This is useful for phylogenetic inference. The translation program can obtain the amino acid sequences according to multiple genetic codes. In addition to the batch processing, it can also read single FASTA file or single DNA sequence (manual mode), thus providing a simple way to get the amino acid sequences. Alignment construction is one of the most important manipulations of sequences and the program can make use of three popular aligners for it: MUSCLE (Edgar, 2004), MAFFT (Katoh et al., 2002), and PRANK (LÖytynoja & Goldman, 2005). The first two programs can generate final alignments quickly and automatically recognize the type of sequence (DNA or amino acid). Although PRANK is slower than the others, it produces more accurate results (Jordan & Goldman, 2012) and can directly obtain final alignments at the codon-level. In addition, FasParser can convert alignments to different formats, for example from FASTA to PHYLIP, PAML, or NEXUS. Batch processing of these functions only needs a directory containing all the sequence files to be analyzed.

## SEQUENCE COMPARISON AND MUTATION IDENTIFICATION

After constructing an alignment, it is often desirable to visualize the mutations or substitutions between two sequences, and/or identify overlapping regions generated by different aligners for the same gene. The programs "Cmp-2Seq" and "Cmp-2Align" address these issues. Cmp-2Seq counts and displays differences between two sequences at the levels of nucleotides and codons. Under the codon level, the program estimates the total number of sites with synonymous (S) and non-synonymous (N) substitutions for the first sequence and then calculates the number of synonymous and non-synonymous substitutions between the two sequences according to the NG86 method of Nei & Gojobori (1986). This function is useful in analyses, such as cancer genomic studies that focus on understanding the selective pressures following cell proliferation (Liu et al., 2012).

**Figure 2:**
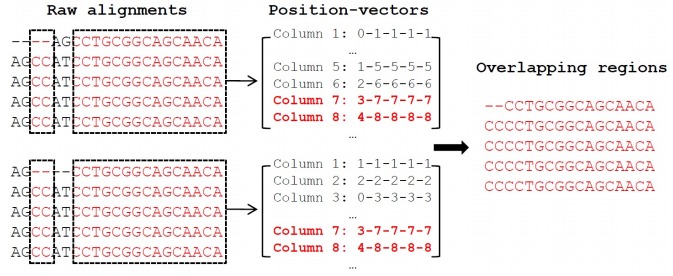
Algorithm used to compare different alignments

Cmp-2Align identifies overlapping regions between two alignments using a simple but rigorous algorithm (Figure 2). Briefly, for each base of an alignment column, the program calculates its gap-free position in the raw sequence. Next, it transforms these positions to a string vector, like "1-2-2", meaning there are 3 sequences, and this column contains the first base of the first sequence, the second base of the second sequence and also the second base in the third sequence. Finally, the program extracts all columns with the same position-vectors between two alignments (Figure 2). This manipulation is useful for analyses such as the identification of regions informative for phylogenetic inference.

## EXTRACTION AND FILTRATION

FasParser can also extract and filter a set of sequences from a raw FASTA file ("Fas-Filter") based on query IDs, as well as removing sequences according to a cutoff-length. Fas-Filter can cut a raw alignment by removing columns with gaps based on a cutoff value of gap frequency. Moreover, the program can also provide summary-statistics of a raw alignment, such as pointing out one or more too short sequences and calculating the length of gap-free blocks.

## COMPARISONS BETWEEN FASPARSER WITH OTHER PROGRAMS

The FasParser package provides a graphic user interface (GUI) with several commonly used functions that perform sequence manipulations. This package remains limited in that it cannot perform phylogenetic inference, edit alignments and identify open reading frames (ORF) (Table 1). Therefore, FasParser is not a replacement of other packages, such as MEGA. Nonetheless, new functions to FasParser are in the process of development.

**Table 1:** Comparisons between FasParser with other programs

	MEGA	BioEdit	FasParser
GUI	√	√	√
Batch processing	×	×	√
Sequence viewing	√	√	√
Alignment comparison	×	×	√
Translation	√	√	√
format conversion	√	√	√
Extraction & filtration	×	√	√
Sequence editing	√	√	×
Phylogeny inference	√	√	×
ORF identification	×	√	×

## ACKNOWLEDGEMENTS

Special thanks to Prof. Robert W. Murphy, Dr. Adeniyi Charles Adeola and Lotanna Micah Nneji for the modifications of this manuscript, and also our colleagues for their suggestions on the improvement of FasParser.
